# Comparison of Surface Proteomes of Adherence Variants of *Listeria Monocytogenes* Using LC-MS/MS for Identification of Potential Surface Adhesins

**DOI:** 10.3390/pathogens5020040

**Published:** 2016-05-17

**Authors:** Hung King Tiong, Steven D. Hartson, Peter M. Muriana

**Affiliations:** 1 Department of Animal Science, Oklahoma State University, Stillwater, OK 74078, USA; htiong@ostatemail.okstate.edu; 2 Robert M. Kerr Food & Agricultural Products Centre, Oklahoma State University, Stillwater, OK 74078, USA; 3 Department of Biochemistry and Molecular Biology, Oklahoma State University, Stillwater, OK 74078, USA; steven.hartson@okstate.edu

**Keywords:** orbitrap, *Listeria monocytogenes*, adherence, adhesin, protein

## Abstract

The ability of *Listeria monocytogenes* to adhere and form biofilms leads to persistence in food processing plants and food-associated listeriosis. The role of specific surface proteins as adhesins to attach *Listeria* cells to various contact surfaces has not been well characterized to date. In prior research comparing different methods for surface protein extraction, the Ghost urea method revealed cleaner protein content as verified by the least cytoplasmic protein detected in surface extracts using LC-MS/MS. The same technique was utilized to extract and detect surface proteins among two surface-adherent phenotypic strains of *L. monocytogenes* (*i.e.*, strongly and weakly adherent). Of 640 total proteins detected among planktonic and sessile cells, 21 protein members were exclusively detected in the sessile cells. Relative LC-MS/MS detection and quantification of surface-extracted proteins from the planktonic weakly adherent (CW35) and strongly adherent strains (99-38) were examined by protein mass normalization of proteins. We found that *L. monocytogenes* 99-38 exhibited a total of 22 surface proteins that were over-expressed: 11 proteins were detected in surface extracts of both sessile and planktonic 99-38 that were ≥5-fold over-expressed while another 11 proteins were detected only in planktonic 99-38 cells that were ≥10-fold over-expressed. Our results suggest that these protein members are worthy of further investigation for their involvement as surface adhesins.

## 1. Introduction

The cytoplasmic membrane and cell wall layers in Gram-positive bacteria are composed of phospholipids and peptidoglycan, respectively, where both serve as attachment platforms for surface proteins [1,2]. The localization of surface proteins is governed by their signal peptide and various forms of interaction with the cell layers [3,4,5]. Surface proteins surrounding the bacterial cell may be produced constitutively or facultatively, and may respond to signaling stimuli in the environment. They may aid in transporting organic substrates or inorganic factors, help to secrete metabolic products, assist in attaching to biotic and abiotic surfaces, form biofilms, and help in escaping host intracellular mechanisms of innate immunity [4,6,7]. Surface proteins also provide virulence functions in pathogenic Gram-positive bacteria such as autolysin (AtlE) corresponding to polystyrene adherence of *Staphylococcus epidermidis*, hemolysin (LLO/Hly) involved with phagocytic membrane cytolysis by *L. monocytogenes*, and pyruvate oxidase (SpxB) for hydrogen peroxide resistance in *Streptococcus pneumoniae* [8,9,10,11].

Identifying proteins has never been more sensitive, less laborious, and easier due to the availability of improved analytical technologies and biochemical kits [2,12]. However, sensitive analytical detection may be compromised by contaminating proteins/peptides introduced as artifacts during experimental processes, as has been observed when using lithium chloride, tris-buffered urea, and trypsin surface-shaving surface protein extraction methods whereby these methods allow cell leakage of cytoplasmic proteins [13,14,15,16,17]. The Matrix Assisted Laser Ionization Time-of-Flight Mass Spectrometer (MALDI-TOF MS) analyzes individually-isolated proteins. However, recent advances linking liquid chromatography separation to mass spectrometry has produced powerful proteomic tools (*i.e.*, orbitrap LC-MS/MS) capable of making simultaneous identifications of proteins in complex mixtures [12,18].

Genomic databases are available for *in silico* identification of proteins with homolog function and homology identity of surface-associated proteins involved with pathogenesis. This facility enhances functional genomics by providing insight into pathogenic mechanisms using comparative protein homology studies. To date, many pathogenic protein determinants, separately involved in biofilm formation, cellular adhesion to biotic and abiotic surfaces, internalization in host cells, cellular escape from host defense mechanisms, and proteins involved in survival towards stress conditions have been identified in minimally characterized pathogenic bacteria using such computer-based search tools [3,19,20,21,22,23,24]. The aim of this study was to exploit cell surface proteomic techniques to identify and quantify recovered pure protein members extracted from the cell surface [16]. Surface-extracted proteins were identified by comparative analysis of cell surface proteomes recovered from strongly and weakly adherent phenotypic variants of *L. monocytogenes* using a gel-less approach of 2D nanoliquid chromatography coupled with an ion-trap mass spectrometry (2DnLC-MS/MS orbitrap) mass analyzer.

## 2. Results and Discussion

### 2.1. Adherence Analysis and Molecular Typing of *L. Monocytogenes* CW35 and 99-38

**Figure 1:**
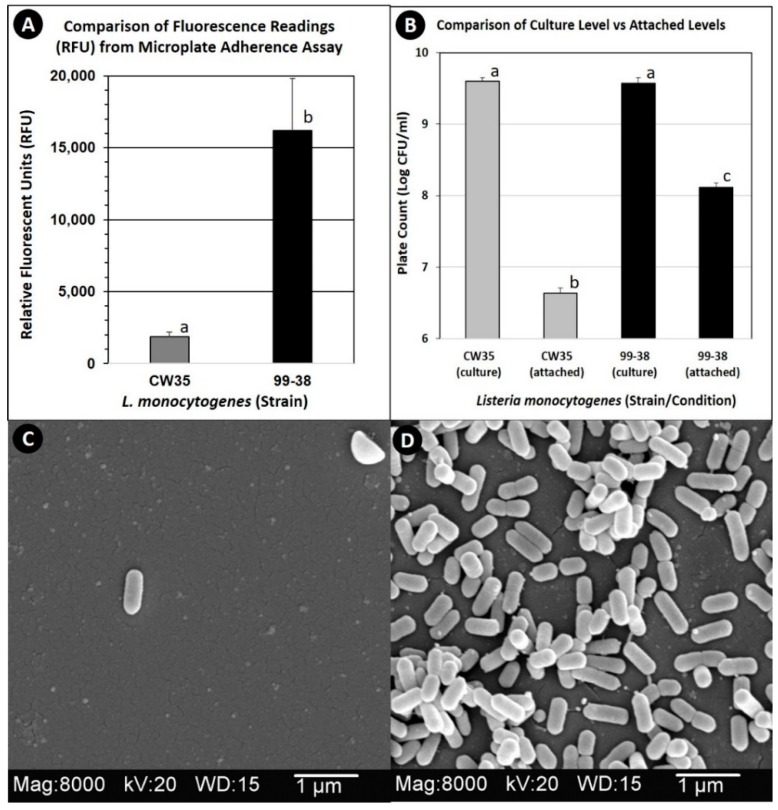
Comparison of weakly adherent *L. monocytogenes* CW35 and strongly adherent *L. monocytogenes* 99-38 by microplate fluorescence adherence assay (panel **A**), enzymatic detachment from biofilms on microplates (panel **B**), and scanning electron microscopy (panels **C** and **D**: CW35 and 99-38, respectively). Graphical data represent the average of triplicate replications and error bars represent the standard deviation from the mean. Bars with the same lowercase letter are not significantly different (*P* > 0.05); bars with different lowercase letters are significantly different (*P* < 0.05).

**Figure 2:**
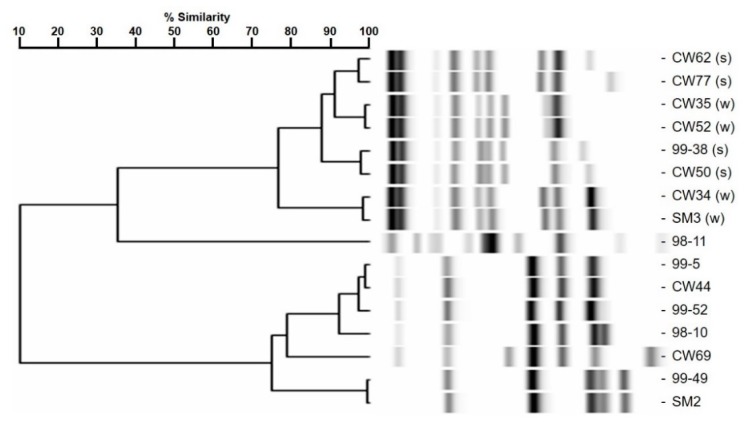
RiboPrint patterns and dendrogram analysis of relatedness for various strains of *L. monocytogenes*, notably CW35 and 99-38 (‘s’ and ‘w’ refer to strong and weak adherence, respectively).

**Figure 3:**
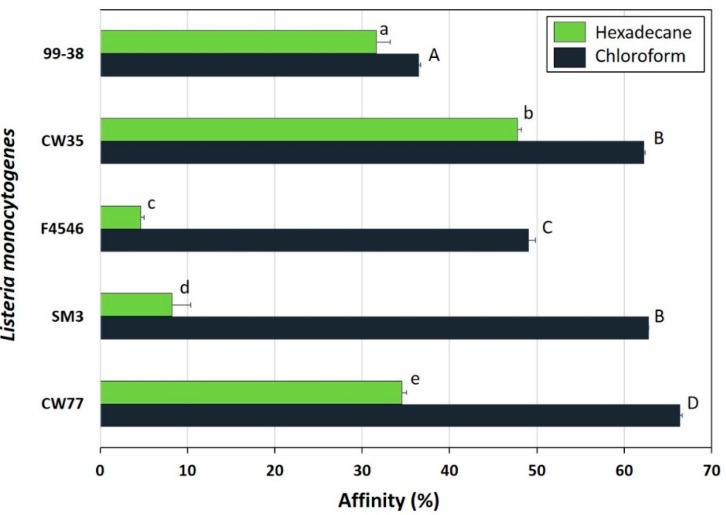
Hydrophobic affinity assays for strains of *L. monocytogenes*. High values indicate hydrophobic tendencies; low values indicate non-hydrophobic (hydrophilic) tendencies. Data bars represent the mean of duplicate samples and replications, and error bars represent the standard deviation from the mean; ‘s’ and ‘w’ refer to strong or weak adherence. Within a solvent grouping, data bars with the same lowercase or uppercase letter are not significantly different (*P* > 0.05); bars with different lowercase or uppercase letters are significantly different (*P* < 0.05).

*L. monocytogenes* 99-38 was confirmed as a strongly adherent strain using the same microplate assay that was originally used to characterize multiple strains in our collection by showing significantly higher relative fluorescence levels than the weakly adherent CW35 strain (Figure 1A). In spite of using the same cell culture levels (~10^9^ cfu/mL) to initiate attachment, strain 99-38 demonstrated a 30-fold greater level of cellular attachment than strain CW35 (Figure 1B). Scanning electron microscopy (SEM) analysis under similar conditions of attachment as in the microplate adherence assays also confirmed low adherence yields by strain CW35 (Figure 1C), while demonstrating an abundance of adhered cells of strain 99-38 (Figure 1D). Genotyping by RiboPrint patterns indicate that although strains CW35 and 99-38 (~86% similarity) do not show identical typing patterns, other strains show a greater disparity in genotype comparison (Figure 2). Serotype examination by multiplex PCR demonstrated that *L. monocytogenes* CW35 typed to serogroups 4b, 4d, and 4e, and strain 99-38 typed to serogroup 1/2a, 3a, both from lineage I (data not shown). Examination of cellular solvent affinity using polar (chloroform) and non-polar (hexadecane) solvents was inconclusive as strain CW35 (weakly adherent) showed a greater propensity to partition into the non-polar/hydrophobic phase than the strongly adherent 99-38 strain (Figure 3). Also, both adherence variant strains showed the least partitioning disparity between polar/non-polar solvents compared to other strongly or weakly adherent strains tested (Figure 3).

### 2.2. Total Surface Protein Identification

#### 2.2.1. Planktonic Cells

**Figure 4:**
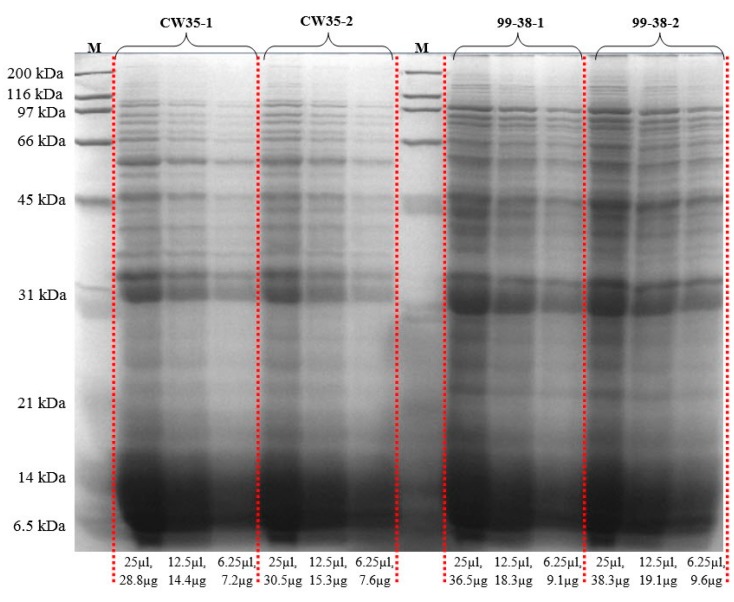
Comparative protein profiles from subcellular proteins prepared by the UB-Ghost protein extraction method examined by 1D SDS-PAGE. Extracts from two biological replications from weakly adherent (CW35) and strongly adherent (99-38) strains of *Listeria monocytogenes.* Protein marker, M; protein amounts below the figure are for total protein loaded per lane.

**Figure 5:**
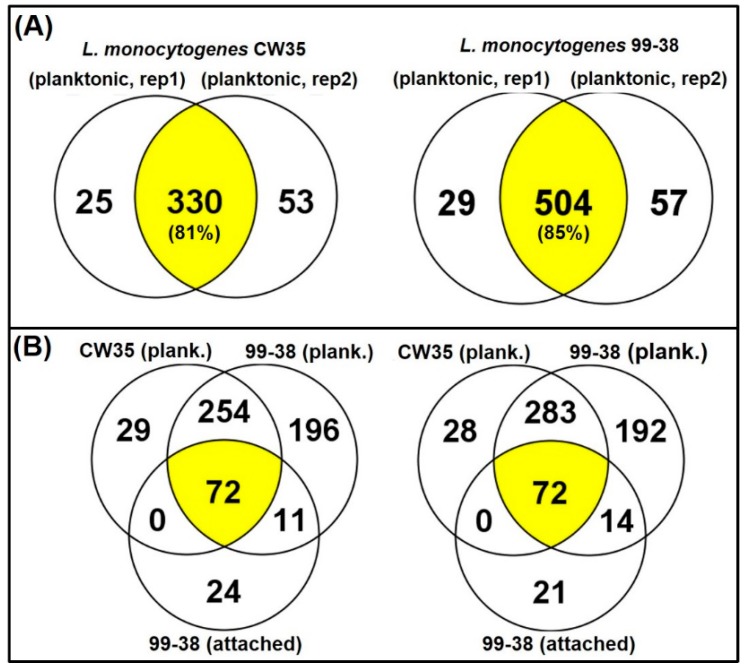
Venn diagrams of identified protein distributions among strains (*i.e.*, CW35, 99-38), biological reps (rep 1 & 2), and cell treatments (planktonic *vs.* attached). Panel **A**, comparison of proteins between biological replications of extractions with *L. monocytogenes* CW35 (**left**) and 99-38 (**right**). Panel **B**, three-way comparison of proteins identified from planktonic cells of CW35, planktonic cells of 99-38, and attached cells of 99-38 in biological replication 1 (**left**) and 2 (**right**). Each biological replicate represents the average of triplicate technical/analytical replications.

The adherence of *Listeria* on equipment and food contact surfaces is one of the factors for the high incidence of contamination of foods produced in ready-to-eat (RTE) food manufacturing facilities. Previously, our lab differentiated various adherent phenotypes among strains of *L. monocytogenes* isolated from raw/processed meats and RTE meat processing facilities and classified them as weakly, moderately, or strongly adherent [25]. We have deployed these isolates as a platform into studies on the molecular basis of adherence in *L. monocytogenes* leading to its persistence in food processing environments. Analysis of the published genome of the type strain, *L. monocytogenes* EGD-e [26], revealed a total of 605 protein species associated with the cell wall (132), membrane (335), and secretions (138). However, only three adherence-associated surface proteins (lmo0433, lmo0434, and lmo0435) involved with attachment to abiotic surfaces have been validated to date in this pathogen [27,28,29]. In the current study, we applied the Ghost urea method for surface protein extraction that reduces contamination by cytosolic proteins, normalized samples based on total protein content (Figure 4), and performed peptide identification by high resolution LC-MS/MS (orbitrap) [16]. We identified a total of 619 protein species in the recovered protein extracts from planktonic cells of two *L. monocytogenes* food isolates, *L. monocytogenes* 99-38 (strongly adherent; 590 proteins) and CW35 (weakly adherent; 408 proteins) and categorized them as surface proteins or cytosolic proteins based on predicted localizations using online prediction tools described previously [16]. The 619 proteins detected from both planktonic strains represents ~22% of the protein species predicted from genomic analysis of the type strain, *L. monocytogenes* EGD-e (2846 gene coding sequences) [30]. These proteins were repeatedly detected in three separate analyses performed on each of two independently prepared surface extracts (biological reps) from planktonic cells. These replications gave reproducibility rates of 81% and 85% in detecting the same proteins between biological reps for CW35 and 99-38, respectively (Figure 5; Table S1). A total of 92 protein species (of the 619 total detected) exhibited a 99-38 (planktonic)/CW35 (planktonic cells) relative total spectrum count ratio of ≥5-fold, in which 11 protein species were detected in extracts from both strongly adherent planktonic and sessile cells (Table 1) while the remaining 81 species were detected in planktonic cells alone (Supplemental Table S1), of which 11 protein species demonstrated a 99-38/CW35 peptide ratio of ≥10-fold relative expression (Table 2).

#### 2.2.2. Sessile Cells

Enrichment of sessile cells was carried out by sub-culturing the strongly adherent *L. monocytogenes* strain 99-38 in media containing glass beads to increase surface attachment area and then extracting proteins from washed adhered cells. A total of 107 proteins were identified, of which 21 proteins were exclusively found in sessile/attached cells and not detected in planktonic surface protein extracts (Table S1).

### 2.3. Envelope Protein Identification

A total of 124 protein species detected among all cell preparations, including 99-38 planktonic (109 proteins), CW35 planktonic (67 proteins), and 99-38 sessile (14 proteins) surface extracts, were categorized according to the *Listeria* genome database, ListiList [31,32] (Table 4, Supplemental Table S1). The ListiList functional category “cell envelope and cellular processes” (code 1) includes subcodes for cell wall (1.1), transport/binding (1.2), signal transduction (1.3), membrane bioenergetics (1.4), mobility (1.5), secretion (1.6), cell division (1.7), cell surface (1.8), and cell transformation (1.10). Proteins responsible for signal transduction (1.3) and cell transformation (1.10) were not detected in all cell extracts. This may be due to low abundance of competence-related proteins due to DNA prophage insertions [47], the fact that cell signaling proteins may be membrane bound, or the result of phase-dependent gene regulation [48,49]. Soluble internalin (1.9) was not detected in all cell extracts and may be attributed to the effective washing process used prior to and during surface protein extraction, hence producing much cleaner surface extracts. Unlike the study of Calvo *et al.* [2], this work identified a total of 38 protein species responsible for ListiList cell wall (28 in this study *vs.* 4 in Calvo) and surface (10 in this study *vs.* 15 in Calvo) in planktonic surface protein extracts that are separately responsible for invasion, metabolism, cell envelope biosynthesis, penicillin binding, peptidase, transportation, and rod shape determination. A smaller number of such protein species (cell wall, 5; cell surface, 4) were detected in sessile surface extracts, indicating enrichment of specific proteins in sessile incubated cells that may be responsible for adhesion, invasion, motility, and cell wall biosynthesis. It is noteworthy that the ListiList-identified cell wall proteins lmo0275 (lmo0275, DNA uptake, not validated), and lmo0394 (P60-like invasion protein homolog, not validated) and the cell surface proteins lmo0204 (ActA, cell-to-cell motility), lmo0434 (InlB, adhesion and invasion) [50], and lmo2713 (internalin-like protein, not validated) were exclusively detected in surface extracts from sessile cells (Table 3), as compared with the more abundant planktonic protein extracts in this study and as observed by Calvo *et al.* [2].

To date, only three surface adhesins responsible for *Listeria* attachment to abiotic surfaces have been identified. Of these, two are known *Listeria* invasins of human hosts (internalins A and B) [27,28]. The data mentioned above suggest that bifunctional (‘moonlighting’) proteins may be involved in the process of *Listeria* attachment to abiotic surfaces *and* in virulence. Recently, Piercey *et al.* [51] attributed regulatory functions to internalins A and B, further strengthening the involvement of a protein in multiple functions such as virulence, attachment, and biofilm formation. Bae *et al.* [52] demonstrated that a *L. monocytogenes* locus *lcp* (*Listeria* cellulose binding protein, LCP) may be involved with binding to carbohydrates on the surface of both host cells and vegetables. Quorum sensing may also be a mechanism by which attachment to select surfaces regulates the expression of certain genes that impact both adherence and virulence. For instance, toxin production by *Staphylococcus aureus* is pronounced when adhered, but not when in a planktonic state [53]. This may be explained by the ready availability of secreted self-regulating molecules on neighboring high-density adhered cells as opposed to dilution of secreted regulatory molecules among low-density/scattered planktonic cells.

Among all the ListiList cell wall and surface protein species identified in this study, the gene expression levels of many of them have been shown to be upregulated (lmo0434, lmo2713, lmo0204), downregulated (lmo2505, lmo2691), or neutral (lmo2558) when investigated by intracellular infection assay [50,54] (Table 5, Supplemental Table S1). *In silico* analysis of all the identified ListiList envelope proteins for detection of surface-associated signal peptides such as LPXTG/NXZTN [33], GW [55], Lipoprotein [36], SecA [44], and TM [36] by web-based tools revealed that among planktonic/sessile surface extracts, 0/0, 11/1, 0/0, 13/0, 6/0, 28/8, and 57/5 protein species were envelope proteins bearing LPXTG/NXZTN, GW, LIPO, SecA, TM, multiple signals, and unknown signal pathway, respectively (Table 6).

### 2.4. Protein Subcellular Localization Prediction

A total of 503 and 93 protein species were detected in extracts from planktonic (99-38: 481; CW35: 341) and sessile (99-38: 93) cells were designated as non-envelope associated proteins by the *Listeria* genome database using multiple protein subcellular localization tools to analyze for prediction of cell surface-associated proteins. These tools included Leger, LocateP, Psort, CW-PRED, LIPO-PRED, transmembrane segment, SignalP, TAT-PRED, and hydropathy plot in which each of them detects a specific surface-associated signal peptide in a protein for prediction of surface protein localization [16]. A total of 389 surface proteins, identified and distributed among sessile (82) and planktonic (377) surface proteins (non-ListiList surface proteins), were mainly ribosomal and hypothetical/unknown proteins, respectively, and are represented in the ListiList functional category (see Supplementary Table S1).

Moonlight proteins have been identified in bacteria, including *L. monocytogenes*, as proteins demonstrating multiple locations and functions [52,57,58,59,60,61,62,63]. A number of such proteins include the gene product responsible for the ListiList functional group elongation (lmo1657; lmo2653, translation elongation factor) [64], main glycolytic pathways (lmo2455, enolase) [65], and specific pathways (lmo1634, alcohol acetaldehyde dehydrogenase) [66]. Burkholder *et al.* [67] reported a surface localization pathway (*i.e.*, SecA2) used by *Listeria* cells to localize lmo1634 on the cell surface. In addition, Chen and others documented the attachment of InlA and InlB surface adhesins/invasins to an abiotic contact surface (*i.e*., glass) [27,28]. These studies confirm multiple locations and functions for some proteins. So many moonlighting proteins have been identified that a database has been established [68].

In our study, both the lmo1634 and lmo2653 gene products were detected in all cell surface extracts (planktonic and sessile) with the total spectrum count ratio of planktonic extracts 99-38/CW35 <2-fold. Other main glycolytic pathway proteins, including glyceraldehyde-3-phosphate dehydrogenase (lmo2459), were identified in all surface extracts. A ribosomal protein adhesin homolog such as L12 (RplL), reported in *Neisseria gonorrhoeae* [69], was exclusively detected in the *L. monocytogenes* (lmo0251) planktonic surface extracts (99-38 and CW35) in this study. However, nothing is known about the adherence-related functions of lmo2459, lmo0251, and the rest of the ListiList functional categories (Table 5). Remarkably, this work exhibited cytoplasmic proteins that were either surface-associated with known or unknown signal peptide, but 16S rRNA proteins were not detected in the extracts, suggesting that the extraction method was reasonably effective in eliminating intracellular proteins.

Variation in the ListiList cell surface and cell wall protein species detected in this work was comparable to the findings reported by Calvo *et al.* [2] and differences may be attributed to different incubation temperatures (30 °C *vs.* 37 °C) as reported by Gorski *et al.* [70], McGann *et al.* [71], and Peel *et al.* [72]. Calvo *et al.* [2] reported a positive correlation between temperature and mRNA levels of *inl*A/*inl*B in a member of *L. monocytogenes* 1/2a, which is commonly associated with foodborne illness outbreaks. McGann *et al.* [71] observed increased attachment levels (fewer planktonic cells were recovered and hence a lower plate count) in unspecified strains of *L. monocytogenes* pregrown at higher temperatures, and applied separately to radish tissue as compared to the same strains pregrown at lower temperatures, suggesting that different molecular factors were involved in attachment at various temperatures. Peel *et al.* detected increased levels of flagellin when incubated at temperatures <37 °C. Altogether, the various conditions used explain the different protein findings among these separate groups of researchers in the identification of surface protein species.

## 3. Experimental Section

### 3.1. Bacterial Cultures and Growth Conditions

Bacterial cultures were obtained from our laboratory culture collection. *L. monocytogenes* CW35 was isolated from retail RTE frankfurters [73] and *L. monocytogenes* 99-38 was isolated from retail raw ground beef. These strains were previously characterized in microplate adherence assays in comparison to other strains isolated from meat processing plants as weakly or strongly adherent, respectively, to abiotic surfaces [25,74]. Subsequent studies evaluating their interaction with tissue culture cells [75] or live mouse assay [76] demonstrated that the strongly adherent form was more invasive than the weakly adherent phenotype. Bacterial cultures were inoculated into brain heart infusion broth (BHI, Difco, Detroit, MI, USA) and incubated overnight at 30 °C and transferred twice before use in experiments.

### 3.2. Strain Characterization: Adherence Assays, Electron Microscopy, Molecular Typing, Serotyping, and Cellular Hydrophobicity

Microplate fluorescence assay and quantification of adhered cells. The microplate fluorescence assay was performed as described earlier [25,74]. Briefly, culture cells in fresh BHI broth media were incubated overnight at 30 °C in 96-well microplates (Nunc, Denmark), aspirated and washed with a microplate washer (ELx405 Magna, BioTek Instruments, Winooski, VT, USA), replenished with sterile BHI broth and incubated again for another cycle of growth and washed again to remove loose, planktonic cells. A fluorescent substrate (5,6-carboxyfluorescein diacetate; 5,6-CFDA) was added to the cells attached to the wells and incubated for 15 min to allow absorptive uptake and cytoplasmic hydrolysis of the substrate. The microplates were washed again on the plate washer to remove external fluorescent substrate and then read on a GENios fluorescence microplate reader (Phenix Research Products, Hayward, CA) with excitation at 485 nm and detection at 535 nm. Attached bacterial cell levels were confirmed by proteolytic release from attached surfaces as described previously [74]. Fluorescence and adherence data were analyzed by one-way analysis of variance (ANOVA) using the Holm-Sidak test for pairwise multiple comparisons to determine significant differences (*P* < 0.05) using the software program SigmaPlot 13.0 (SPSS Inc., Chicago, IL, USA). In prior studies, neither the fluorescent substrate (5,6-CFDA) nor the proteolytic treatment affected the viability of cells as determined with cells in liquid culture.

Scanning electron microscopy. Overnight cultures were diluted in sterile BHI broth and incubated in eight-well CultureSlides (Becton-Dickinson, Franklin Lakes, NJ, USA). The glass slides were washed and submitted for scanning electron microscopy (SEM) analysis by technicians at the Oklahoma State University Electron Microscope core facility.

Molecular typing. Bacterial strains were genotyped using the RiboPrinter Molecular Characterization System (DuPont Qualicon, Wilmington, DE, USA) to generate ribotypes using the restriction enzyme, *Eco*R1 and following the manufacturer’s instructions. A phylogenetic tree was developed based on cluster analysis of data by the unweighted pair group method with arithmetic averages (UPGMA).

Serotyping. Serogroup determination of *L. monocytogenes* CW35 and 99-38 was performed by multiplex PCR as described by Doumith *et al.* [77] in comparison to strains of known serotype (*L. monocytogenes* EGDe and V7, 1/2a; ScottA, 4b). Genomic extraction was prepared on washed, overnight cells by the bead collision method [78] and PCR reactions were performed as multiplex PCR reactions with primer sets of lmo0137, lmo1118, ORF2110, ORF2819, and prs as described [77]. Amplicons were examined on 2% agarose gels after staining with EtBr for documentation.

Microbial adherence to solvent (MATS) assay for cell surface hydrophobicity characterization of *Listeria* strains. Microbial adherence to solvent is based on the surface hydrophobicity of cell envelope components [79]. Briefly, a suspension of 10^8^ cfu/mL bacterial cells (prewashed 3x in the same NaCl) in 2.4 mL of 0.15 M NaCl were mixed with 0.4 mL of a solvent (chloroform or hexadecane) with a vortex mixer. The homogenate was allowed to form two phases, and 1 mL of the aqueous phase was removed for absorbance reading at 400 nm. The percentage of microbial affinity to a solvent is evaluated using a formula as follows: 100 × [1 − (AS/A0)] = % Affinity; A_0_ indicates the optical density at 400 nm of the cell suspension (without solvent added) and A_S_ is the absorbance of the aqueous phase of the homogenate (with solvent added). Pairwise multiple comparisons for statistical significance (*P* < 0.05) were performed for data within each group of solvents by ANOVA using SigmaPlot 13.0 (SPSS), as described above.

### 3.3. Extraction of Surface Proteins from *Listeria Monocytogenes*

Cells grown in broth. The UB-Ghost method for extracting surface proteins was derived from previous protocols reported by Boone *et al.* [80] and Cordwell [81] and modified by Tiong *et al.* [16]. Cells were grown overnight at 30 °C in 50 mL BHI broth (Difco). Pelleted cells were incubated for 1 h at 4 °C with 600 μL of sterile nanopure water containing 5 mM EDTA (USB Co., Cleveland, OH, USA), with agitation at 1200 rpm (Pulsing Vortex Mixer, VWR Intl., Atlanta, GA, USA). After incubation, the cells were pelleted by gentle centrifugation (3000× *g*, 6 min, 4 °C), decanted, and the cell pellet was washed three times in 10 mL of 10 mM phosphate-buffered saline (PBS, pH 7.4). After three washes, the final pellet was resuspended in 100 µL 8 M urea buffer and incubated for 30 min at ambient temperature with agitation at 3000 rpm. After incubation in urea buffer, the cells were pelleted by gentle centrifugation (3000× *g*, 4 °C, 6 min) and the supernatant was collected, filtered through 0.45 μm syringe filters (Pall Newquay, Cornwall, UK), and stored at −80 °C.

Cells adhered to glass beads. In an initial overnight incubation with glass beads, *L. monocytogenes* was incubated overnight (~20 h) at 30 °C in screw cap tubes containing 60 mL BHI broth and 80 g of 5-mm diameter soda lime glass beads (VWR Scientific). The spent culture was removed and replaced with 10 mL of fresh BHI broth and the beads were allowed to slowly turn on a rotisserie for 10 min before being replaced again with another 10 mL of fresh BHI broth; after the third replacement with fresh BHI, the tubes were allowed to incubate statically overnight. This process was repeated daily for seven days to promote attachment and enrich for attached cells. After seven days, the spent broth was removed and cells (attached to glass beads) were washed three times (10 min each) in 1x PBS (10 mL, pH 7.4) on a rotisserie; this washing procedure was then repeated with sterile nanopure water containing 5 mM EDTA. The cells/beads were washed one last time by incubating for 1 h at 4 °C in 10 mL sterile water on the rotisserie and the wash fluid was then discarded. Surface proteins of *L. monocytogenes* attached to glass beads were then extracted by rotating the glass beads with 10 mL buffered urea solution (8 M urea, pH 8.5, 5 mM EDTA, 5 mM β-mercaptoethanol) for 45 min at ambient temperature. The recovered buffered urea solution with extracted proteins was centrifuged (12,000 rpm, 4 °C) to remove contaminating cells and filter sterilized using a 0.45-µm filter. Proteins in the extracted buffered urea solution were precipitated with absolute ethanol (1 volume protein extract: 4 volumes absolute ethanol) [82]. Air-dried ethanol-precipitated protein samples were rehydrated in 8 M urea containing 5 mM EDTA and 5 mM β-mercaptoethanol.

### 3.4. Protein Concentration Measurement

Protein extracts were quantified using the Pierce bicinchoninic acid (BCA) protein assay kit (ThermoFisher Scientific, Waltham, MA, USA) combined with the reducing agent compatible (RAC) reagent, as instructed by the manufacturer. Absorbance readings were obtained with a Genesys 20 spectrophotometer at a wavelength of 280 nm (ThermoFisher).

### 3.5. SDS-PAGE Analysis

Protein extracts were resolved on 1-D SDS-PAGE gels (12.5%) for visual validation and run overnight at 50 volts in an SE600 Vertical Electrophoresis System (Hoefer, Holliston, MA, USA), as described by Laemmli [83]. Protein extracts were loaded at three different concentrations (1x, 2x, 4x) from 7.2–38.3 μg per well (or lane). The resolved proteins were stained with Coomassie blue R-250 for visualization (Figure 4). 

### 3.6. Orbitrap Mass Spectrometry (LC-MS/MS)

Protein samples, accompanied by SDS-PAGE analyses and concentration readings, were analyzed for protein identities and quantities at the Oklahoma State University DNA/Protein Core Facility. A hybrid LTQ-OrbitrapXL mass spectrometer (Thermo Fisher Scientific) coupled to a New Objective PV-550 nano-electrospray ion source and an Eksigent NanoLC-2D chromatography system (Eksigent, Framingham, MA, USA) was used. Protein sample digestion, liquid chromatography, and MS analyses were performed as previously described [16,84] with minor modifications. Briefly, protein fragments were prepared by overnight trypsinization of protein samples in the presence of denaturing (urea), reducing (Tris-2-carboxyethyl-phosphine), and alkylating (iodoacetamide) agents before subjected to liquid chromatography and tandem mass spectrometry (LC-MS/MS). Proteome samples were normalized against total protein by physical loading of equal amounts of protein (0.4 μg of total protein) into the column [85,86,87]. All reagents were prepared in a Tris-HCl buffer. Peptides were analyzed by using chromatography columns packed with 20 cm of 3-micron Magic C18 AQ particles (Bruker) and eluted using a 3%–34% acetonitrile gradient over a period of 105 minutes.

### 3.7. MS Data Analysis, Protein Identification, and Proteomic Analysis

Ion masses were used to identify proteins, as described previously, with minor modifications [16,76]. Briefly, searches were performed with Mascot (Matrix Science, London, UK; v. 2.2.04) and X! Tandem (thegpm.org; CYCLONE, ver. 2010.12.01.1) [88] using the *L. monocytogenes* EGD-e database (5939 protein sequences), downloaded from NCBI on 01/26/11, and supplemented with 112 sequences of common protein contaminants. Mascot and X! Tandem were searched with fragment ion mass and parent ion tolerances of 0.80 Da and 5.0 PPM, respectively. An allowance of max missed cleavage numbers of 1 and 2 was set for Mascot and X! Tandem, respectively. The searches also included parameters for variable peptide modifications elicited by pyroglutamate cyclization of N-terminal glutamines, oxidation of methionine, acylation of cysteine by acrylamide and iodoacetamide adducts, and formylation and acetylation of the protein N-terminus.

The Scaffold program (Proteome Software, Portland, OR, USA; ver. 2.2.00) and the Peptide Prophet Algorithm were used for validation of peptide/protein identities and construction of Venn diagrams. The identifications were conducted with a protein threshold of 95%, two minimum peptides, and a peptide threshold of 80% [89]. Proteins that contained similar peptides and could not be differentiated based on MS/MS analysis alone were grouped to satisfy the principles of parsimony. The observed protein false discovery rate (FDR) rate was one percent. Additional tools for proteomic analysis (ListiList, LocateP, PSORT, Cell Wall Predictor, Lipoprotein Predictor, Transmembrane Prediction, SignalP Identification, Hydropathy, and GRAVY values) were used as described previously [16].

### 3.8. Statistical Test for Determining Significant Differential Expression

The Fisher Exact Test [90,91] was performed on spectral count data collected by Multidimensional Protein Identification Technology (MudPIT) analysis of bacterial surface protein digests to validate the significant difference of comparative quantification by total spectrum counts. The significant threshold and difference, *p*-values, were generated using Scaffold (Proteome Software).

## 4. Concluding Remarks

To our knowledge, the current work reveals the first comparative identification of total surface proteins detected in surface extracts of two different adherence phenotypes of *L. monocytogenes* (strongly-adherent 99-38 and weakly-adherent CW35). The key purification step in the UB-Ghost method involved bleeding off cytosolic components prior to surface protein extraction. We consider this as critical for enhanced recovery of surface proteins, as opposed to other proteomic standard protocols involving trypsin, LiCl, or urea extraction buffer where cytosolic contaminants are readily present in the resulting extract [16]. A greater variety of surface-associated proteins identified by LC-MS/MS were represented by moonlight proteins (389), ListiList “envelope and cellular processes” (124), and analyses of surface proteins, as reported by other groups [2,92,93].

Different protein species as well as total spectrum counts were detected in surface extracts of the *L. monocytogenes* adherence variants enriched by planktonic or sessile conditions. A higher number of protein species was detected in the planktonic cell surface extracts (619) as compared with the sessile extract (107). This could be explained by the greater abundance of planktonic cells grown in culture media than sessile cells attached to glass beads. In spite of this imbalance, a group of five ListiList envelope protein species (lmo0275, lmo0394, lmo0204, lmo0434, and lmo2713) were exclusively detected in the 99-38 *L. monocytogenes* surface extract enriched by sessile incubation, as compared with the planktonic extracts in this study or the Calvo group [2]. This may indicate that there were specific protein factors required for sessile activities such as biofilm formation, cell wall maintenance, and cell attachment.

Among the protein species identified, a total of 141 surface-associated proteins (ListiList and non-ListiList, identified by subcellular localization tools) were without signal peptides, suggesting that other signal determinants of protein surface transportation may yet be discovered. Currently, there is no singular all-in-one, up-to-date protein localization tool that includes biochemically validated locations of proteins. For instance, lmo0202 (hly) [50] and lmo1634 (lap) [66,67] have been validated as surface proteins for years but this information has not been updated in the *Listeria* genome database, ListiList [32], or Leger [26,31]. As a result, many web-based protein localization tools were deployed in this study and hence the process was time-consuming and complicated. This information is useful in functional characterization of protein homologs in close bacterial relatives and expedites targeted analysis of virulence factors such as surface adhesins in foodborne pathogenic bacteria.

We feel the data presented herein offer compelling validation towards the use of LC-MS/MS to the detection of bacterial surface proteomes that are expressed under select conditions. Our data show a difference in expression on abiotic surfaces (glass beads) relative to planktonic cells and their adherence phenotypes and warrant further studies in this area. We suspect that additional adhesins may be involved/expressed when *L. monocytogenes* is attached to vegetable surfaces and hope to apply similar methods for extracting surface proteins from *L. monocytogenes* directly attached to produce. These are important food safety issues as more *Listeria* outbreaks have been linked to fruits and vegetables that are often consumed without cooking.

## Figures and Tables

**Table 1:** Isolated surface proteins detected in both attached and planktonic cells where expression fold change of planktonic cells 99-38 (strong)/CW35 (weak adherence) ≥5-fold, or not present in planktonic cells CW35**);** data are the average of three technical replicates for each of two biological replicates. C: cell wall, CY: cytoplasm, CM: cytoplasmic membrane, E: Extracellular, M: membrane, S: secreted, TM: transmembrane, TMH: transmembrane helix. A star (*) indicates a relative significant difference of total spectrum count between the same proteins detected in planktonic 99-38 and CW35 cells with a *P*-value threshold of <0.02.

Gene (ListiList) ^a^	Protein Function Homolog (kDa)	Leger ^b^	LocateP ^c^	Psort ^d^	CW-PRED ^e^	PRED-LIPO ^f^	Trans ^g^	SignalP ^h^	PREP-TAT ^i^	Hydropathy Score ^j^	GRAVY Score ^k^	Total Spectrum Count Detected by LC-MS/MS		
												99-38 Attached	99-38 Plank.	CW35 Plank.
lmo0199 * (2.3)	Phosphoribosyl pyrophosphate synthetase (35)	M	CY	0.03 (E,C) CY	No	No	0	No	No	<1.8	−0.03	4	15	2
lmo0415 * (2.1.1)	Endo-1,4-beta-xylanase (52)	CY	M	0.33 (E,C,CM) E	No	Yes (TM)	1	No	Yes (TM)	>1.8	−0.41	3	17	0
lmo0978 * (2.2)	Amino acid aminotransferase (37)	CY	CY	2.5 (CM,E,C) CY	No	No	0	No	No	<1.8	−0.22	4	9	2
lmo1067 * (3.7.4)	GTP-binding elongation factor (69)	M	CY	1.22 (CY,E,C) CM	No	No	0	No	No	<1.8	−0.39	5	16	3
lmo1072 * (2.1.2)	Pyruvate carboxylase (128)	M/S	CY	2.5 (CM,E,C) CY	No	No	0	No	No	<1.8	−0.26	4	22	1
lmo1325 * (3.7.3)	Translation initiation factor IF-2 (85)	CY	CY	0.03 (E,C) CY	No	No	0	No	No	<1.8	−0.45	3	15	1
lmo1504 * (3.7.2)	Alanyl-tRNA synthetase (98)	S	CY	0 CY	No	No	0	No	No	<1.8	−0.37	7	24	4
lmo1519* (3.7.2)	Aspartyl-tRNA synthetase (66)	M	CY	0 CY	No	No	0	No	No	<1.8	−0.29	6	8	0
lmo1663 * (2.2)	Asparagine synthetase (72)	M	CY	2.5 (CM,E,C) CY	No	No	0	No	No	<1.8	−0.43	4	6	0
lmo2558 * (1.8)	Autolysin amidase (102)	C/M/S	Sec	0.02 (C) E	No	Yes (Sec)	1	Yes	Yes (Sec)	<1.8	−0.48	4	70	1
Lmo2608 (3.7.1)	30S ribosomal protein S13 (14)	CY	CY	0.03 (E,C) CY	No	No	0	No	No	<1.8	−0.75	11	5	1

^a^ ListiList functional/location classification code [31,32]. ^b^ *Listeria*’s post-genome database (LEGER) [26]; Updated information to genome database ListiList, agreed upon by the *Listeria* Genome European Consortium [31]. ^c^ LocateP [33] Distinguish cytoplasmic from other subcellular proteins by identifying the no-N-terminal signal sequence: tat/sec. ^d^ PSORTb v3.0.2 protein subcellular localization prediction tool; values represent surface localization score [34]. ^e^ Cell wall predictor by Fimereli, 2012 [35]. ^f^ Lipoprotein predictor by Bagos 2008 [36]. ^g^ Transmembrane segment/helix v. 2.0 prediction [37,38,39]. ^h^ SignalP identification of secreted protein by identifying signal peptide and cleavage site [40,41,42,43]. ^i^ Sec and TAT driven secretion system [44,45]; ^j^ Hydropathy plot [46]; Gravy values = Negative indicates hydrophilic protein, >1.8 at window size 19 indicates transmembrane region in a protein, <1.8 at window size 9 indicates surface protein [46]; ^k^ Average gravy calculator (http://www.gravy-calculator.de/).

**Table 2:** Isolated surface proteins found in planktonic 99-38 cells but not in attached 99-38 cells where the 99-38/CW35 ratio ≥10-fold (average of three technical replicates for each of two biological replicates from planktonic cells). C: cell wall, CY: cytoplasm, CM: cytoplasmic membrane, E: Extracellular, M: membrane, S: secreted, TM: transmembrane, TMH: transmembrane helix. A star (*) indicates a relative significant difference of total spectrum count between the same proteins detected in planktonic 99-38 and CW35 cells with a *P*-value threshold of <0.02.

Gene (ListiList) ^a^	Protein Function (kDa)	Leger ^b^	LocateP ^c^	Psort’s Protein Localization ^d^	CW-PRED ^e^	PRED-LIPO ^f^	Trans ^g^	SignalP ^h^	PREP-TAT ^i^	Hydropathy Score ^j^	GRAVY Score ^k^
lmo0220 * (1.7)	ATP-dependent zinc metalloprotease (76)	C/M	M	0.01 (CY) CM	No	Yes (Lipo)	2	Yes	Yes (Sec)	>1.8	−0.37
lmo0392 * (5.2)	Hypothetical protein (34)	M	M	2.5 (CM,E,C) CY	No	Yes (TM)	1	No	No	<1.8	0.16
lmo0723 * (1.5)	Methyl-accepting chemotaxis protein (66)	CY	M	0.04 (CY) CM	No	Yes (TM)	2	No	Yes (Sec)	>1.8	−0.2
lmo1068 * (6)	Hypothetical protein (31)	M/S	Extracellular (Lipid anchored)	(Equal score to all) Unknown	No	Yes (Lipo)	0	Yes	Yes (Sec)	>1.8	−0.67
lmo1076 * (1.1)	Autolysin (64)	S	Sec	0.02 (C) E	No	Yes (Sec)	1	Yes	Yes (Sec)	>1.8	−0.61
lmo2033 * (1.7)	Cell division protein (46)	M	CY	2.5 (CM,E,C) CY	No	No	0	No	No	<1.8	−0.06
lmo2157 * (5.2)	Hypothetical protein (71)	CY	CY	2.5 (CM,E,C) CY	No	No	0	No	No	<1.8	−0.35
lmo2206 * (4.1)	Clp protease subunit B (98)	M	CY	0.03 (E,C) CY	No	No	0	No	No	<1.8	−0.37
lmo2414 * (2.2)	Aminotransferase (48)	C,M	CY	2.5 (CM,E,C) CY	No	No	0	No	No	<1.8	−0.23
lmo2510 * (1.6)	Preprotein translocase subunit (95)	M	CY	0.03 (EC) CY	No	No	0	No	No	<1.8	−0.46
lmo2748 * (5.2)	Hypothetical protein (16)	CY	CY	2.5 (CM,E,C) CY	No	No	0	No	No	<1.8	−0.40

^a^ ListiList functional/location classification code [31,32]. ^b^ *Listeria*’s post-genome database (LEGER) [26]. Updated information to genome database ListiList and agreed upon by the *Listeria* Genome European Consortium [31]. ^c^ LocateP [33]. Distinguish cytoplasmic from other subcellular proteins by identifying the no-N-terminal signal sequence: tat/sec. ^d^ PSORTb v. 3.0.2 protein subcellular localization prediction tool; values represent surface localization score [34]. ^e^ Cell wall predictor by Fimereli, 2012 [35]. ^f^ Lipoprotein predictor by Bagos 2008 [36]. ^g^ Transmembrane segment/helix v. 2.0 prediction [37,38,39]. ^h^ SignalP identification of secreted protein by identifying signal peptide and cleavage site [40,41,42,43]. ^i^ Sec and TAT driven secretion system [44,45]. ^j^ Hydropathy plot [46]. Gravy values = Negative indicates hydrophilic protein, >1.8 at window size 19 indicates transmembrane region in a protein, <1.8 at window size 9 indicates surface protein [46]. ^k^ Average gravy calculator (http://www.gravy-calculator.de/).

**Table 3:** Isolated surface proteins detected in attached 99-38 cells but not in planktonic 99-38 cells (average of three technical replicates for each of two biological replicates from planktonic cells). C: cell wall, CY: cytoplasm, CM: cytoplasmic membrane, E: Extracellular, M: membrane, S: secreted, TM: transmembrane, TMH: transmembrane helix.

Gene (ListiList) ^a^	Protein Function (kDa)	Leger ^b^	LocateP ^c^	Psort’s Protein Localization ^d^	CW-PRED ^e^	PRED-LIPO ^f^	Trans ^g^	SignalP ^h^	PREP-TAT ^i^	Hydropathy Score ^j^	GRAVY Score ^k^
lmo0046 (3.7.1)	30S ribosomal protein S18 (9)	CY	CY	0.33 (E,C,CM) CY	No	No	0	No	No	<1.8	−0.63
lmo0055 (2.3)	Hypothetical protein	CY	CY	0.03 (E,C) CY	No	No	0	No	No	<1.8	−0.25
lmo0186 (5.2)	Hypothetical protein (44)	CY	M	(Equal score to all) Unknown	No	Yes (TM)	1	No	Yes (TM)	>1.8	−0.47
lmo0202 (4.6)	Listeriolysin O precursor (59)	C/M/S	S	0.28 (C,CM,CY) E	No	Yes (Sec)	0	Yes	Yes (Sec)	>1.8	−0.47
lmo0204 (1.8)	Actin-assembly inducing protein (70)	C/M/S	M	CM	Yes	Yes (Sec)	1	Yes	Yes (Sec)	>1.8	−0.80
lmo0214 (3.2)	Transcription-repair coupling factor (135)	CY	CY	0.03 (E,C) CY	No	No	0	No	No	<1.8	−0.33
lmo0241 (5.2)	Hypothetical protein (28)	CY	CY	1.84 (CY,C,E) CM	No	No	0	No	No	<1.8	−0.18
lmo0275 (1.10)	Hypothetical protein (30)	CY	M	(Equal score to all) Unknown	No	Yes (Sec)	0	Yes	Yes (Sec)	>1.8	−0.22
lmo0394 (1.1)	Listeria extracellular P60 protein (25)	CY	S	0.27 (C,CM) E	No	Yes (Sec)	1	Yes	Yes (Sec)	>1.8	−0.33
lmo0434 (1.8)	Internalin B (71)	S	S	0.79 (CM, E) C	No	No	1	No	Yes (sec)	>1.8	−0.46
lmo0707 (1.5)	Flagellar hook-associated protein 2 FliD (validated) (46)	CY	CY	0.28 (C,CM,CY) E	No	No	0	No	No	<1.8	−0.33
lmo0724 (5.2)	27 Hypothetical protein (27)	CY	M	0.45 (CY,C,E) CM	No	Yes (Sec)	1	No	Yes (Sec)	>1.8	−0.17
lmo1272 (5.2)	Ribosomal biogenesis GTPase (33)	CY	CY	1.84 (CY,C,E) CM	No	No	0	No	No	<1.8	−0.44
lmo1480 (3.7.1)	30S ribosomal protein S20 (9)	CY	CY	0.33 (E,C,CM) CY	No	No	0	No	No	<1.8	−0.83
lmo1784 (3.7.1)	50S ribosomal protein L35 (8)	CY	CY	0.33 (E,C,CM) CY	No	No	0	Yes	No	<1.8	−1.22
lmo2156 (5.1)	Hypothetical protein (13)	S	M	(Equal score to all) Unknown	No	Yes (Sec)	1	Yes	Yes (Sec)	>1.8	−0.44
lmo2505 (1.1)	Peptidoglycan lytic protein P45 (43)	C/M/S	S	0.27 (C,CM) E	No	Yes (sec)	1	Yes	Yes (Sec)	<1.8	−0.55
lmo2619 (3.7.1)	30S ribosomal protein S14 (7)	CY	CY	0.33 (E,C,CM) CY	No	No	0	No	No	<1.8	−0.61
lmo2656 (3.7.1)	30S ribosomal protein S12 (validated) (15)	C/M	CY	0.03 (E,C) CY	No	No	0	No	No	<1.8	−0.76
lmo2691 (1.1)	Autolysin, N-acetylmuramidase (64)	C/S	M	2.18 (C,CM) E	No	Yes (Sec)	1	No	Yes (Sec)	<1.8	−0.74
lmo2713 (1.8)	GW repeat-containing cell wall binding repeat protein (35)	CY	S	0.13 (C,E) CM	No	Yes (Sec)	0	Yes	Yes (Sec)	>1.8	−0.54

^a^ ListiList functional/location classification code [31,32]. ^b^ Listeria’s post-genome database (LEGER) [26]. Updated information to genome database ListiList agreed upon by the Listeria Genome European Consortium [31]. ^c^ LocateP [33]. Distinguish cytoplasmic from other subcellular proteins by identifying the no-N-terminal signal sequence. ^d^ PSORTb v. 3.0.2 protein subcellular localization prediction tool; values represent surface localization score [34]. ^e^ Cell wall predictor by Fimereli 2012 [35]. ^f^ Lipoprotein predictor by Bagos 2008 [36]. ^g^ Transmembrane segment/helix v. 2.0 prediction [37,38,39]. ^h^ SignalP identification of secreted protein by identifying signal peptide and cleavage site [40,41,42,43]. ^i^ Sec and TAT driven secretion system [44,45]. ^j^ Hydropathy plot [46]. Gravy values = Negative indicates hydrophilic protein, >1.8 at window size 19 indicates transmembrane region in a protein, <1.8 at window size 9 indicates surface protein [46]. ^k^ Average gravy calculator (http://www.gravy-calculator.de/).

**Table 4:** Functional classification of surface proteins identified by LC-MS/MS (orbitrap).

Code–Functional Group ^a^	Attached *Listeria* Strain 99-38	Planktonic *Listeria* Strain 99-38	Planktonic *Listeria* Strain CW35	*Listeria* Strain EGD-e
1—Cell envelope; cellular processes	14 (2.3%) ^b^	109 (17.6%)	67(13.1%)	620
2—Intermediary metabolism	26 (4.3%)	152 (24.9%)	94 (18%)	611
3—Information pathways	59 (13.1%)	139 (30.8%)	106 (26.1%)	452
4—Other	2 (1.3%)	34 (22.8%)	23 (18.1%)	149
5—Similar to unknown proteins	6 (0.8%)	136 (18.2%)	100 (14.3%)	746
6—No similarity	0	20 (7.7%)	18 (8.1%)	260
Total	107(3.8%)	590 (20.8%)	408 (14.4%)	2838

^a^ Functional groups of proteins were assigned according to the classification codes of the *Listeria* genome and the LEGER proteome databases [26,31,32]. ^b^ The number or proteins identified and the percentage relative to total EDG-e proteins in each code category.

**Table 5:** Cell envelope proteins (surface proteins) expressed in planktonic cells at a 99-38/CW35 peptide ratio ≥ 10-fold, or found in attached cells, identified by ListiList^1^ or protein localization tools. A star (*) indicates a relatively significant difference of total spectrum count between the same proteins detected in planktonic 99-38 and CW35 cells with a *P*-value threshold of <0.02.

#	Gene ID	Gene Name	ListiList Functional Category	ListiList Code	Presence in Sessile Cells	Expression in Planktonic Cells	*In Vivo* Regulation (literature) ^a,b^
1	lmo0002 *	dnaN	DNA replication	3.1	---	>10	---
2	lmo0055	purA	Metabolism of nucleotides and nucleic acids	2.3	√	---	---
3	lmo0186	lmo0186	Unknown	5.2	√	---	---
4	lmo0202	hly	Miscellaneous	4.5	√	---	↑
5	lmo0204 ^1^	actA	Cell surface proteins protein precursor	1.8	√	---	↑
6	lmo0241	lmo0241	Unknown	5.2	√	---	---
7	lmo0275 ^1^	lmo0275	Transformation/competence	1.10	√	---	---
8	lmo0394 ^1^	lmo0394	Cell wall	1.1	√	---	---
9	lmo0434 ^1^	inlB	Cell surface proteins	1.8	√	---	↑
10	lmo0705 *^,1^	lmo0705	Mobility and chemotaxis	1.5	---	>10	---
11	lmo0707 ^1^	lmo0707	Mobility and chemotaxis	1.5	√	---	---
12	lmo0724	lmo0724	Unknown	5.2	√	---	---
13	lmo0898 *	lmo0898	Unknown	5.2	---	>10	---
14	lmo1072 *	pycA	Main glycolytic pathways	2.1.2	√	>10	Neut
15	lmo1272	lmo1272	Unknown	5.2	√	---	---
16	lmo1325 *	infB	Initiation (translation)	3.7.3	√	>10	---
17	lmo1360 *	folD	Metabolism of coenzymes and prosthetic groups	2.5	---	>10	---
18	lmo1544 *^,1^	minD	Cell division	1.7	---	≥10	↓
19	lmo1699 *^,1^	lmo1699	Mobility and chemotaxis	1.5	---	>10	---
20	lmo1784	rpmI	Ribosomal proteins	3.7.1	√	---	---
21	lmo1860	msrA	Protein modification reductase A	3.8	---	≥10x	---
22	lmo1953	pnp	Metabolism of nucleotides and nucleic acids	2.3	---	>10	---
23	lmo2156	lmo2156	Unknown	5.1	√	---	---
24	lmo2415 *^,1^	lmo2415	Transport/binding proteins and lipoproteins	1.2	---	>10	---
25	lmo2505 ^1^	spl	Cell wall	1.1	√	---	↓
26	lmo2525 *^,1^	mbl	Cell wall	1.1	---	≥10	---
27	lmo2558 *^,1^	ami	Cell surface proteins	1.8	√	≥10	Neut
28	lmo2656	rpsL	Ribosomal proteins	3.7.1	√	---	---
29	lmo2691 ^1^	lmo2691	Cell wall	1.1	√	---	↓
30	lmo2713 ^1^	lmo2713	Cell surface proteins	1.8	√	---	↑

NA: No information available in the literature; ^a^ Chatterjee *et al.*, 2006 [54]; ^b^ Camejo *et al.*, 2011 [50].

**Table 6:** Distribution of different signal types of the ListiList envelope protein species identified by LC-MS/MS (orbitrap).

Surface Extract from:	LPXTG/NXZTN ^a^	GW ^b^	LIPO ^c^	Sec ^d^	TM ^c,d^	GW & Sec	GW, LIPO & Sec	LIPO & Sec	Sec & TM	GW & TM	LPXTG, GW, Sec	LPXTG & Sec	Unknown Signal	Total
Planktonic cells	0	11	0	13	6	8	5	8	3	1	1	2	57	115
Sessile cells	0	1	0	0	0	1	2	5	0	0	0	0	5	14

^a^ LPXTG was identified by LocateP [33]. ^b^ GW domain identification tool [55,56]. ^c^ Lipoprotein predictor by Bagos 2008 [36]. ^d^ Sec and TAT driven secretion system [44,45].

## Supplementary Materials

The following are available online at www.mdpi.com/2076-0817/5/2/40/s1. Table S1: Complete MS Spectrum Count Data.
